# CCD Pixel Miniaturization Technologies for Spatial Resolution Enhancement

**DOI:** 10.3390/s26165077

**Published:** 2026-08-10

**Authors:** Zhenyu Liu, Xiaowei Lyu, Zizhuo Liu, Hao Xu

**Affiliations:** 1 School of Physics, University of Electronic Science and Technology of China, Chengdu 611731, China; 2 Institute of Fundamental and Frontier Sciences, University of Electronic Science and Technology of China, Chengdu 611731, China

**Keywords:** charge-coupled device, image sensor, high resolution, pixel miniaturization

## Abstract

Charge-coupled device (CCD) image sensors stand out for their high sensitivity, low noise, and strong radiation tolerance, which have established them as core components in high-end imaging fields such as astronomical observation, remote sensing, and defense and security. With increasingly stringent requirements for imaging system performance in emerging applications, including ultra-high resolution, intelligent sensing, and multimodal information fusion, CCD technology continues to evolve toward higher levels of integration and improved performance. Within a fixed sensor area, one of the most straightforward ways to raise image resolution is to shrink pixel size, that is, CCD pixel miniaturization. In this review, we provide a systematic survey of the major advances in CCD pixel miniaturization achieved in recent years. We first detail four mainstream CCD architectures—linear-array, full-frame, frame-transfer, and interline-transfer—along with several newly developed architectures, and compare their performance characteristics and application constraints. We then summarize the key technologies that enable pixel miniaturization and examine several closely related technological directions. Finally, we look at the current state of CCD pixel miniaturization and discuss its future development trends.

## 1. Introduction

Since its invention by Willard S. Boyle and George E. Smith at Bell Laboratories in 1969, the Charge-Coupled Device (CCD) has become one of the most important semiconductor image sensing technologies [1,2,3]. Owing to its superior performance, including high sensitivity, low readout noise, low dark current, and wide dynamic range, CCDs have consistently played a vital role in high-end imaging applications [4]. A CCD functions by collecting photo-generated carriers and transferring them via charge coupling, which enables efficient photoelectric conversion while keeping signal distortion to a minimum. With progress in fabrication processes and design strategies—such as backside-illuminated architectures, deep-depletion structures, and pinned photodiodes—the quantum efficiency (QE) and spectral response of CCDs have been substantially improved. Although consumer-grade CCDs reached a pixel size of approximately 1.3 µm around 2011, the performance requirements for scientific-grade and industrial-grade CCDs differ from those for consumer-grade CCDs; therefore, the CCDs mentioned above all have relatively large pixel sizes. Considering the current situation of CCDs in the consumer imaging market, this article mainly focuses on the application of CCDs in science and industry.

Notably, CCDs can retain advantages over complementary metal-oxide-semiconductor (CMOS) image sensors in scenarios requiring low-light sensitivity, high dynamic range, and precision measurement. The most significant difference between CCD and CMOS lies in the fact that CCD transfers the charge from all pixels column by column before performing circuit processing, whereas in CMOS, each pixel has its own amplifier that processes the signal first. This gives CCD relatively higher image quality and lower noise, while CMOS offers faster processing speed and low power consumption. With advances in lithography technology, the image quality of CMOS has improved substantially. Benefiting from lower power consumption, higher integration, and compact component design, CMOS has attracted considerable attention and investment, and now dominates the vast majority of the consumer market. However, CCD still retains certain advantages in fields such as near-infrared detection, time-delay integration (TDI), and integrated imagers. In near-infrared detection, the epitaxial layer of CCD can be made thicker, and the circuit and doping concentration adjustments are also more flexible. For TDI devices, CCD has a natural advantage in summing signal charges, whereas CMOS tends to accumulate noise when performing pixel-level charge summation on TDI devices. Therefore, CCD is often the preferred choice for TDI applications where high speed is not a critical requirement. Moreover, the high image quality and low noise characteristics of CCD have also spurred widespread interest and research into the CCD-in-CMOS process [5,6,7]. CCD image sensors continue to maintain an irreplaceable technological position and widespread adoption in professional and scientific applications requiring high-quality imaging, such as astronomical observation, aerospace remote sensing, military reconnaissance, and biomedical imaging, owing to their excellent immunity to interference, superior signal-to-noise ratio (SNR), high dynamic range, and outstanding linearity. As application scenarios continue to expand and become increasingly complex, fields such as aerospace, defense security, and scientific exploration have placed more stringent requirements on the performance of imaging systems. In particular, driven by cutting-edge applications including small-satellite remote sensing, high-resolution Earth observation, astronomical survey observations, and precision guidance, image sensor technologies are rapidly evolving toward ultra-high spatial or spectral resolution, enhanced radiation-hardened performance, highly intelligent information extraction, and multimodal information fusion [8,9,10,11]. Pixel miniaturization in CCDs represents the most direct technological approach to improving the spatial resolution of image sensors. However, as pixel size continues to shrink, the physical and electrical characteristics face multiple challenges under the condition of meeting the detection requirements. First, the reduction in the photosensitive area of each pixel leads to a significant decrease in full well capacity (FWC), thereby limiting the dynamic range of the image and increasing the risk of saturation. Second, the reduced pixel size exacerbates both optical and electrical crosstalk, resulting in signal distortion and degradation of the modulation transfer function. Meanwhile, the shrinking charge-storage section makes the effects of readout noise and dark current noise more pronounced, thereby reducing the SNR of the image. Furthermore, during the charge-coupled transfer process, pixel miniaturization also imposes more stringent requirements on charge transfer efficiency (CTE) and the associated peripheral driving circuitry.

In recent years, major international CCD manufacturers and research institutions have continued to make significant progress in the development of scientific and industrial grade image sensors. With regard to pixel miniaturization, several generations of scientific CCD products developed by Teledyne e2v have featured a range of pixel sizes, among which the CCD-47-20 employs a pixel size of 13 μm and the CCD-290-99 employs a pixel size of 10 μm [12]. CCD products developed by Hamamatsu for scientific imaging and high-precision spectroscopic analysis predominantly utilize pixel sizes of 14 μm and 12 μm, while its high-speed readout CCD-S12379 also adopts an 8 μm pixel size [13]. Oxford Instruments has developed CCD and Electron-Multiplying CCD (EMCCD) cameras with pixel sizes ranging from 13 μm to 16 μm [14]. Significant advances have also been achieved by domestic research institutions in the field of CCD image sensors. The 44th Research Institute of China Electronics Technology Group Corporation has successfully developed a high-definition, low-blooming CCD image sensor with a pixel size of 9 μm and an array format of 2048 × 2048, exhibiting excellent anti-blooming and anti-smearing characteristics. In addition, the institute has developed a CCD image sensor featuring a 7.4 μm pixel size and a 1920 × 1080 array format. By employing an amplifier design with a 1.0 μm polysilicon gate length, the device demonstrates significantly enhanced anti-blooming performance under high-illumination conditions [15,16]. In terms of technology integration, the rapid advancement of CMOS fabrication processes has led to the emergence of the CCD-in-CMOS concept, which combines the superior image quality and low-noise characteristics of CCDs with the low power consumption and low manufacturing cost of CMOS technology. This concept has attracted extensive research interest and experimental validation [17,18,19]. Furthermore, emerging two-dimensional (2D) materials have provided new opportunities for CCD development [20]. Research groups at Zhejiang University have explored and developed novel CCDs based on silicon–molybdenum disulfide and silicon–graphene heterostructures [21,22,23,24,25,26]. While remaining compatible with existing integrated circuit fabrication processes, these devices achieve sensitivity and responsivity comparable to those of conventional CCDs at the same pixel size, thereby opening new avenues for the future development of CCD technology. In addition, specialized CCD architectures, such as the Skipper CCD [27,28], have been proposed to address specific application requirements. Although CCDs continue to face challenges in pixel miniaturization, including reduced FWC, increased crosstalk, elevated noise levels, and degraded CTE, which collectively slow the scaling process, domestic research institutions are gradually advancing CCD technology toward higher levels of integration and improved performance through innovative device architectures and material engineering. Table 1 presents the parameters of some of the CCDs mentioned above and the CCDs presented in the table belong to different types.

## 2. Types and Principles of CCD

**Figure 1:**
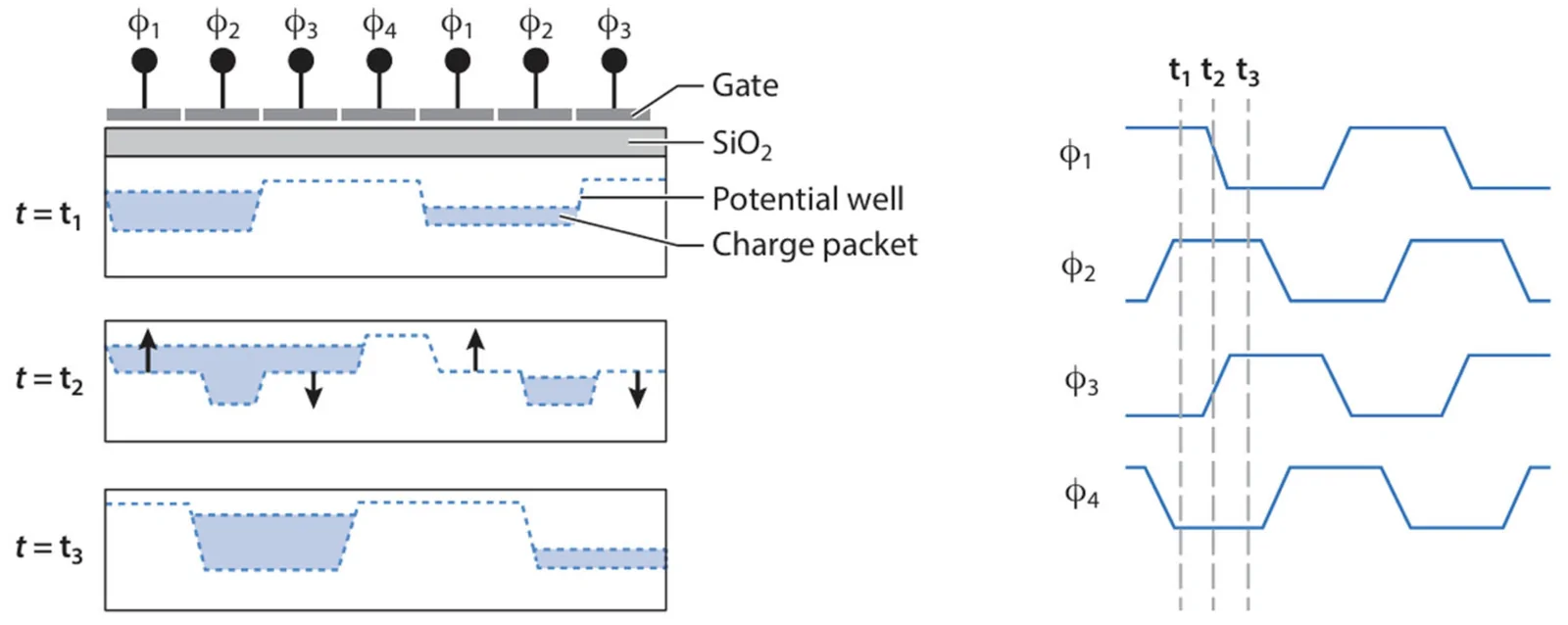
Illustration of a four-phase CCD diagram, a potential well diagram, and clock charts. As four clocks switch sequentially, the potential wells move rightward together with the charge packets (Reproduced from Reference [30] with permission from E.R. Fossum, et al., Annual Review of Vision Science; published by Annual Reviews, 2024).

A CCD is a type of semiconductor charge transfer device. Its fundamental structure consists of an array of densely packed metal–oxide–semiconductor capacitors fabricated on a semiconductor substrate, with adjacent capacitors isolated by continuous insulating layers [29]. Under the action of externally applied clock pulses, depletion regions are formed beneath the CCD gate electrodes, where minority carriers generated through the photoelectric effect are collected and stored in potential wells, forming discrete charge packets. By controlling the voltages applied to neighboring gates according to a specific timing sequence, these charge packets can be transferred directionally along a predetermined path, thereby accomplishing signal transport and readout. Illustration of a CCD diagram and clock charts is shown in Figure 1 [30]. Since its emergence in the 1970s, CCD technology has evolved continuously alongside advances in semiconductor fabrication processes and device architectures, becoming a key imaging and sensing technology in numerous scientific and technological fields. To satisfy the diverse requirements of different applications in terms of performance, operating speed, and integration level, a variety of CCD architectures have been developed. During the pixel miniaturization process, different types of CCDs exhibit varying degrees of sensitivity to parameters such as SNR, FWC, crosstalk and so on. Consequently, the required technological and performance improvements also differ. For example, the interline-transfer CCD (IT-CCD) is more significantly affected by optical crosstalk during pixel miniaturization. Therefore, it is necessary to introduce CCDs with different architectures. The following sections provide a systematic overview of several mainstream CCD structural configurations.

### 2.1. Linear CCDs

**Figure 2:**
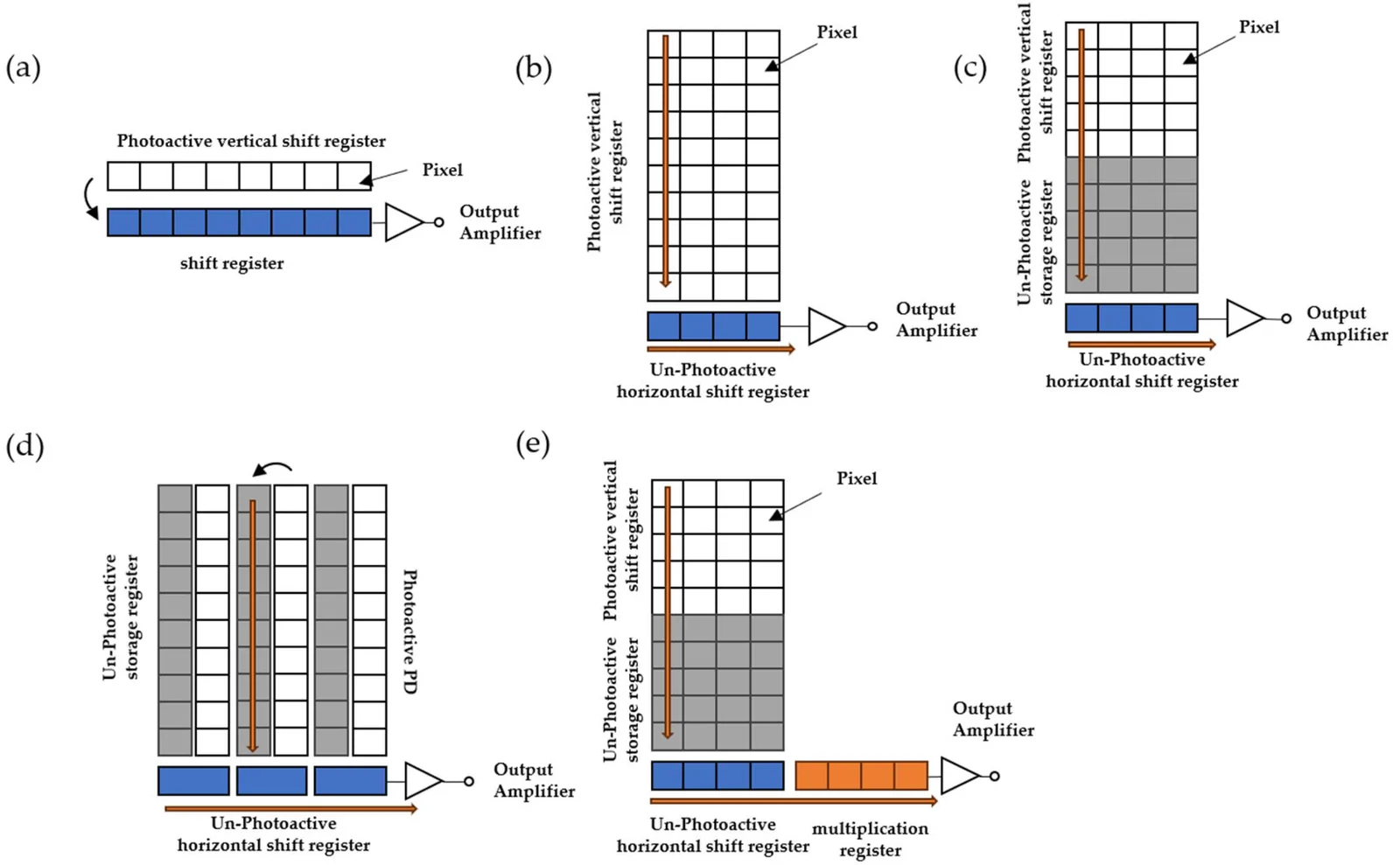
Schematic diagram of various types of CCDs: (**a**) Schematic diagram of a linear CCD charge transfer, where the charge generated by each photoactive pixel is transferred to the shift registers for output; (**b**) Schematic diagram of a full-frame CCD charge transfer, in the image section, charges are collected in the photoactive pixel area. After exposure, charges are sequentially transferred to the un-photoactive storage registers in the storage section and then to the un-photoactive horizontal shift registers for readout; (**c**) Schematic diagram of an FT-CCD charge transfer, where charges in the image section are first transferred to a shielded storage section and then to the readout registers; (**d**) Schematic diagram of an IT-CCD charge transfer, where charges in the image section are first transferred to adjacent shielded storage section and then sequentially transferred to the un-photoactive horizontal shift registers below; (**e**) Schematic diagram of an EMCCD charge transfer, an additional multiplication register is added after the un-photoactive horizontal shift registers to perform electron multiplication before readout.

A linear CCD (Figure 2a) is composed of a single row of pixels, with all photosensitive elements aligned along one straight line [31]. Compared to area-array CCDs, its structure is simpler, it has fewer pixels, and the pixel array occupies a smaller area. Architecturally, the pixel array runs parallel to the shift register, and charge readout is handled serially by just one output register. To speed up readout, a dual-channel scheme is often adopted in practice. Linear CCDs also have no frame storage section, which keeps the structure compact and simple. Because the pixel array is one-dimensional, building a 2D image requires relative movement between the sensor and the target. A mechanical scanning system is therefore typically added to realize 2D imaging. These devices are mainly applied in tasks that need a long field of view and high-precision observation, such as one-dimensional dynamic target measurement, positioning and inspection, and spectral measurement.

### 2.2. Full-Frame CCDs

A full-frame CCD is a traditional and simple area-array CCD architecture in which the entire pixel array is used simultaneously for photo-detection and readout, without a dedicated charge transfer storage section. A shift register network is located beneath the entire array. Due to the absence of an optically shielded storage section, if charge were transferred immediately after exposure while illumination persists, incident light would continue to generate photo charges in the pixels, leading to image smearing. Therefore, a mechanical shutter or a synchronized strobe is required. During the exposure stage, all pixels generate and accumulate charge, which is stored in their respective potential wells, with the exposure time controlled by the shutter. After exposure, the shutter is closed to block incoming light, and charge transfer is then performed, shifting charges row by row into a serial readout register. The readout process is repeated until all row information has been transferred, as shown in Figure 2b. In this process, the clock frequency of vertical transfer determines the row transfer speed; excessively low speeds may lead to charge leakage or crosstalk. Because full-frame CCDs do not include an optically shielded layer, they achieve a larger effective exposure area under the same device footprint, resulting in a very high fill factor, higher imaging sensitivity, and lower noise, making them suitable for high-resolution and low-light imaging applications. However, since this architecture requires the shutter to be closed during charge transfer, it suffers from a very low frame rate and is therefore unsuitable for high-speed dynamic imaging.

### 2.3. Frame-Transfer CCDs

Compared with the full-frame CCD, a frame-transfer CCD (FT-CCD) incorporates an additional optically shielded storage section. Its core architecture consists of two parts: a photoactive section which is also known as the image section and a storage section of the same size as the image section. These two sections are connected through vertical shift registers. The pixel structure of the storage section is identical to that of the image section; however, its surface is covered by a metallic light-shielding layer, preventing photo response and allowing it to serve solely as a temporary charge storage section. The operating cycle of a FT-CCD comprises two stages: frame readout and frame transfer. During the frame readout stage, photo-generated charges are accumulated and stored in the potential wells of the image section, while charges previously stored in the storage section are transferred row by row through the vertical shift registers to the horizontal shift register and subsequently read out serially. Since no mechanical shutter is required, the exposure time is controlled electronically, enabling more flexible exposure adjustment and reducing overall system complexity [32]. During the frame transfer stage, all charge packets in the image section are rapidly shifted into the optically shielded storage section under the control of high-frequency clock pulses applied to the vertical shift register (Figure 2c). This architecture is well suited for high-frame-rate imaging; however, it requires an additional storage section equal in size to the image section, effectively doubling the chip area. Furthermore, instability in the clock signals during rapid charge transfer may lead to charge crosstalk and image smearing between frames, necessitating high-precision driving circuitry for performance optimization.

### 2.4. Interline-Transfer CCDs

The IT-CCD is currently the most widely used area-array CCD architecture. In this structure, optically shielded transfer regions are placed adjacent to segmented photosensitive regions. Each pixel column is divided into two parts: one column serves as the photosensitive region for photoelectric conversion, while the other consists of an optically shielded transfer gate used for temporary charge storage and transfer. After exposure, a driving voltage is applied to the transfer gates adjacent to each photosensitive element, rapidly shifting the accumulated charge from the photosensitive region into neighboring vertical shift register elements. Once the charge is transferred into the vertical shift registers, these registers, controlled by clock pulses, sequentially transfer the charge row by row down to the horizontal shift register located at the bottom of the chip. After receiving charge from all columns, the horizontal shift registers serially output pixel signals to an amplifier, where they are converted into voltage signals, as shown in Figure 2d. During vertical transfer and readout, the photosensitive elements can immediately begin the next exposure, enabling high-frame-rate continuous imaging. This transfer scheme supports continuous operation and increases readout speed; however, it reduces the fill factor and sensitivity of the pixel array and introduces higher fabrication complexity and cost [31]. To mitigate the impact of non-photosensitive areas and improve the fill factor, microlens arrays combined with color filter arrays are typically integrated on each pixel to enhance the efficiency of light collection [30,33]. Furthermore, with the development of Time Delay and Integration (TDI) scanning technology, the inherently lower sensitivity of IT-CCDs can be effectively compensated [34].

### 2.5. Electron-Multiplying CCDs

EMCCD (Figure 2e) introduces a charge multiplication register into the original architecture, enabling signal amplification before readout noise is added [35,36]. As a result, the proportion of readout noise in the total noise of the EMCCD is significantly reduced. While achieving sub-electron readout noise performance, it also exhibits noise characteristics that differ from those of conventional CCDs. It features high resolution, wide dynamic range, high single-photon sensitivity, and high CTE, making it particularly important for low-light imaging applications [37,38].

### 2.6. CCD-in-CMOS Process

During the development of the CCDs, there are many ideas developed in CMOS technologies that can be utilized. Both academia and industry, in the research and design of CCD and CMOS image sensors, have jointly leveraged the advantages of both device types to develop a CCD-in-CMOS process characterized by high integration, low power consumption, and fast readout. These technologies have opened up a new path that differs from the traditional CCDs and have become the research hotspot, although many devices based on new technologies remain at the experimental stage, and their overall performance is currently not as good as that of commercial CCDs.

A common improvement of this process involves modifying the readout architecture on the silicon substrate of conventional CCDs. The charge collection and transmission architecture of the CCD is manufactured through advanced CMOS processes, while the complex image signal acquisition and quantization circuit functions are integrated into the chip, thereby achieving monolithic integration of high image quality and high integration density [5,11]. Moreover, depending on the specific process design, such new technologies hold significant potential in applications requiring high-speed, large FWC, and broadband detection. However, this process also presents certain issues. Some technologies that are mature in CCD and CMOS may not be applicable to this hybrid architecture. For example, the CCD-in-CMOS process abandons the high-voltage overlapping gate operation of conventional CCDs, and the limited voltage leads to a reduction in FWC, which in turn affects dynamic range and image fidelity. Meanwhile, neither traditional CCD nor CMOS simulation models can necessarily be directly applied to simulations; new simulation models need to be developed, which has a certain impact on the design of the new devices [39,40,41].

At present, companies such as Panasonic and Teledyne e2v have introduced multiple prototypes and products based on CCD-in-CMOS architectures, demonstrating excellent performance in fields such as astronomy, X-ray detection, low-light video, and space remote sensing. The CCD-in-CMOS process represents an important development direction for future imaging systems featuring high dynamic range, low noise, and a high degree of integration. In 2016, the Interuniversity Microelectronics Centre in Belgium developed a monolithically integrated, backside-illuminated seven-band TDI CCD-in-CMOS image sensor based on a CMOS process platform. The device features a pixel size of 5 μm, and FWC can reach 18000 electrons while CTE exceeds 99.99% [8].

## 3. Technologies of Pixel Miniaturization

### 3.1. Physical Effects and Performance Trade-Offs in CCD Pixel Miniaturization

CCD pixel miniaturization has several advantages. Smaller pixels can reduce power consumption and weight, improve economies of scale, and lower production costs. For a fixed sensor area, devices with smaller pixels achieve higher resolution. Among the current application fields of CCDs, low-light detection is an important one, and the impact of pixel size reduction on sensitivity in low-light environments is relatively more acceptable.

However, pixel miniaturization also faces significant challenges, such as the impacts on FWC, QE, crosstalk, and noise. The FWC is proportional to pixel area and decreases quadratically as the pixel size shrinks, which leads to a number of issues: the noise within the pixel remains unchanged, resulting in a reduced SNR and dynamic range which is used to quantify a sensor’s ability to image highlights and shadows, and is defined as the ratio of the maximum non-saturated signal to the minimum detectable signal [42]. Moreover, overflow of charges becomes more likely as the FWC decreases, causing crosstalk between adjacent pixels. Smaller pixels impose higher demands on optical components such as microlenses; otherwise, light incident at certain angles cannot be effectively focused, resulting in a reduction of effective light and light entering incorrect pixels, which in turn leads to lower QE and increased crosstalk [43,44]. In addition, for the same array size, smaller pixels mean more pixels, so more transfers, more time, and higher defect rates. For a CCD with a CTE of 0.99999, after 1000 pixel transfers, the overall CTE will be 0.99. The smaller the pixel size, the greater the loss caused by this exponential accumulation. When it comes to the defect rate of CCDs, the combination of increased sensor area and reduced pixel size significantly raises the incidence of hot pixel defects in cameras and sensors [45]. Moreover, the charge transfer characteristics of traditional CCD cause a single defective pixel to affect the entire column of pixels.

Large-pixel detectors offer higher signal-to-noise ratio and dynamic range [46]. If the performance after miniaturization fails to meet the requirements, then miniaturization itself loses its significance. Changing pixel size exerts conflicting effects on key imaging parameters. Therefore, trade-offs must be made during pixel miniaturization, and various techniques should be employed to adjust and optimize these parameters accordingly. The technologies introduced below serve to alleviate these issues. Table 2 shows some technologies of pixel miniaturization, and all of the technologies will be described in the following sections.

### 3.2. Backside-Illuminated Image Sensors

**Figure 3:**
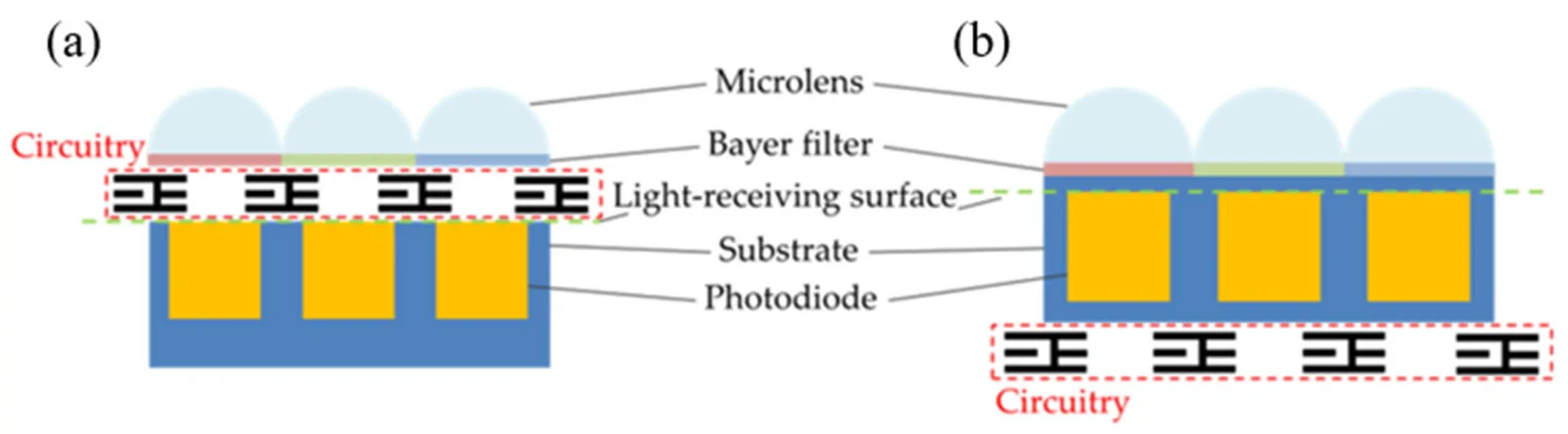
(**a**) Schematic diagram of front-illuminated incidence. (**b**) Schematic diagram of backside-illuminated incidence (Reproduced from Reference [48] with permission from C. Westgate, et al., Sensors; published by MDPI, 2022).

As pixels continue to shrink, each one captures less incident light under the same illumination, which degrades imaging quality. The backside-illuminated design represents one of the most significant and classic improvements in CCDs [47]. A conventional front-illuminated CCD image sensor is illustrated in Figure 3a [48]. With this structure, incident light must travel through complex front-side elements—such as lenses, optical filters, and electrodes—and is then partially absorbed by the polysilicon gate, leading to absorption losses. At the same time, the smooth surfaces of the front-side polysilicon and electrodes reflect visible wavelengths, causing additional loss of incoming optical energy. Ultraviolet and long-wavelength photons also suffer from poor absorption efficiency, giving rise to further transmission losses. All these factors combine to reduce QE [49], and the problem becomes more severe as pixel size decreases. As the push for higher resolution intensifies, demands on both pixel size and pixel count grow stricter, and front-illuminated structures increasingly struggle to meet these requirements.

The backside-illuminated structure is developed from the front-illuminated architecture. A schematic of a device using this structure is shown in Figure 3b. This device offers a significant advantage in terms of QE: the peak QE of backside-illuminated CCDs can exceed 90%, whereas that of front-illuminated CCDs is approximately 40%. The backside-illuminated structure moves structures such as circuitry away from the optical path between the incident light and the photosensitive pixels, thereby reducing losses caused by reflection and refraction during light incidence, significantly improving QE, and lowering crosstalk.

The fabrication process of backside-illuminated devices is more complex and requires several additional steps, including front-side support, backside thinning, backside passivation, and anti-reflection coating processes. Since backside thinning disrupts the silicon lattice and creates many surface-state defects at the backside, even with passivation, it often still raises dark current. Thanks to advances in fabrication technology, backside-illuminated structures have been applied to CCDs and subsequently to CMOS and CCD-in-CMOS devices [50].

### 3.3. Microlenses

The use of microlenses on imaging devices can improve the light collection capability of photoelectric detectors, thereby enhancing the quality of captured images. Thanks to Ishihara, who melted and reflowed resin to form spherical structures [51], enabling mass production of microlenses, such structures have been widely applied in IT-CCDs and other image sensors. Since microlenses can capture more photons from the scene or object, more electron–hole pairs are generated, thereby improving sensitivity and providing more useful information for subsequent signal processing. In the process of pixel miniaturization, the use of microlenses can increase the number of photons per unit area, thereby compensating for the loss caused by pixel size reduction aimed at improving resolution [52,53]. As discussed in Section 2.4, for devices with transfer architectures such as IT-CCD, the impact of pixel size is even more significant. The microlens array is an important optical component of IT-CCD pixels, serving to focus incident light, thereby increasing the fill factor and improving QE. However, if the design of the microlens is not adapted to the device or the application scenario, it may affect light intake and impose certain constraints on photon absorption. When light is incident at a large oblique angle, microlenses may worsen optical crosstalk, so additional measures are required to prevent it.

**Figure 4:**
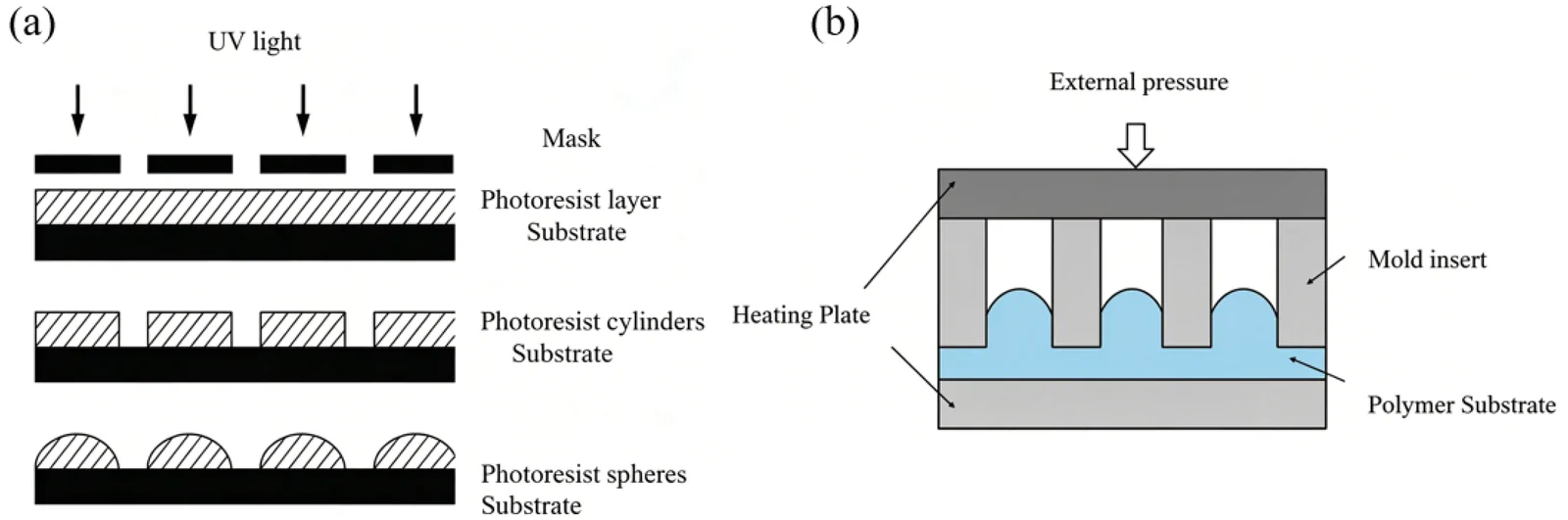
Microlens array fabrication processes: (**a**) thermal reflow method; (**b**) microplastic embossing method (Reproduced from Reference [53] with permission from W. Yuan, et al., Chinese Journal of Mechanical Engineering; published by Springer Nature, 2018).

Microlenses not only require an appropriate focal length to concentrate incident light within the central region of the photosensitive area, preventing light from entering the transfer region and generating smearing signals, but also require optimized surface curvature to increase the effective aperture ratio. In a high-resolution IT-CCD developed by the Chongqing Optoelectronic Technology Research Institute, polymethyl methacrylate (PMMA) was selected as the microlens material [15] and they used thermal reflow method (Figure 4a) to fabricate the microlens array. Apart from the thermal reflow method, there are many other methods for fabricating microlens arrays, such as the microplastic embossing method (Figure 4b) and ultraprecision machining method.

### 3.4. Anti-Blooming Structures

**Figure 5:**
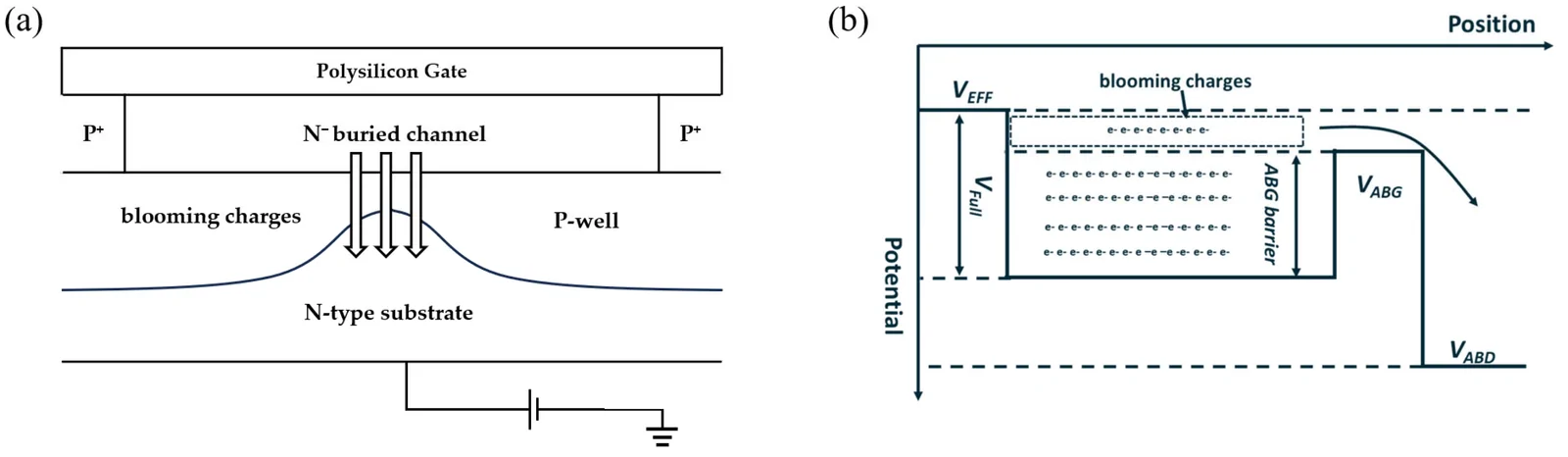
(**a**) Schematic diagram of a vertical anti-blooming structure. (**b**) Schematic of lateral anti-blooming structure potential profile (Reproduced from Reference [11] with permission from S. Guo, et al., Sensors; published by MDPI, 2025).

Another effect of pixel size reduction is the decrease in FWC and the resulting limitation of dynamic range. When the incident light intensity exceeds the saturation level of the CCD, charges exceeding the FWC of a pixel overflow the potential barrier into adjacent pixels, leading to image distortion and blooming artifacts. Anti-blooming structures are designed to reduce crosstalk. Mainstream anti-blooming structures can be classified into two categories: vertical anti-blooming structures and lateral anti-blooming structures, with the distinction mainly depending on the location of the anti-blooming drain. In addition, time-sequenced anti-blooming techniques are also employed [54,55,56,57]. In vertical anti-blooming structures (Figure 5a), for example, an overflow gate is introduced by adding an additional electrode within the pixel. When an anti-blooming bias is applied to the substrate, the saddle-shaped p-well in the photosensitive region is depleted, allowing excess photo-generated carriers to flow through the anti-blooming potential barrier into the substrate, thereby suppressing blooming and preventing charge overflow [58]. Although this structure is not constrained by small pixel size and does not reduce fill factor, it reduces the depth of the photosensitive region, thereby affecting the absorption of long-wavelength light by the sensor, reducing QE of specific wavelength bands and potentially decreasing FWC. In this configuration, photoelectrons generated by long-wavelength photons are directly collected and removed by the vertical anti-blooming drain.

Lateral anti-blooming structures (Figure 5b) suppress blooming by integrating overflow gates within the vertical shift registers to divert excess charge [59]. When the photo-generated signal charge in the photosensitive region exceeds the FWC of the CCD channel, the surplus charge overcomes the anti-blooming potential barrier and is discharged through the overflow drain, thereby achieving blooming suppression [11]. This structure does not affect the absorption of long-wavelength light; however, it occupies part of the effective pixel area of the sensor, thereby reducing the fill factor and FWC. A reduction in FWC will lead to an increase in the noise contribution ratio, thereby affecting SNR and dynamic range of CCDs.

In 2020, a new CCD based on capacitive deep trench isolation (CDTI) was produced. The device is operated with a two-phase structure and the transverse CDTI trenches, which shape electrostatic potential and utilize the vertical axis to develop collection and transfer volumes. Although this technology differs from conventional anti-blooming structures, CDTI also possesses an anti-blooming function. Moreover, the device has many advantages like high FWC, high CTE and nice characteristics on dark current [60,61,62].

**Figure 6:**
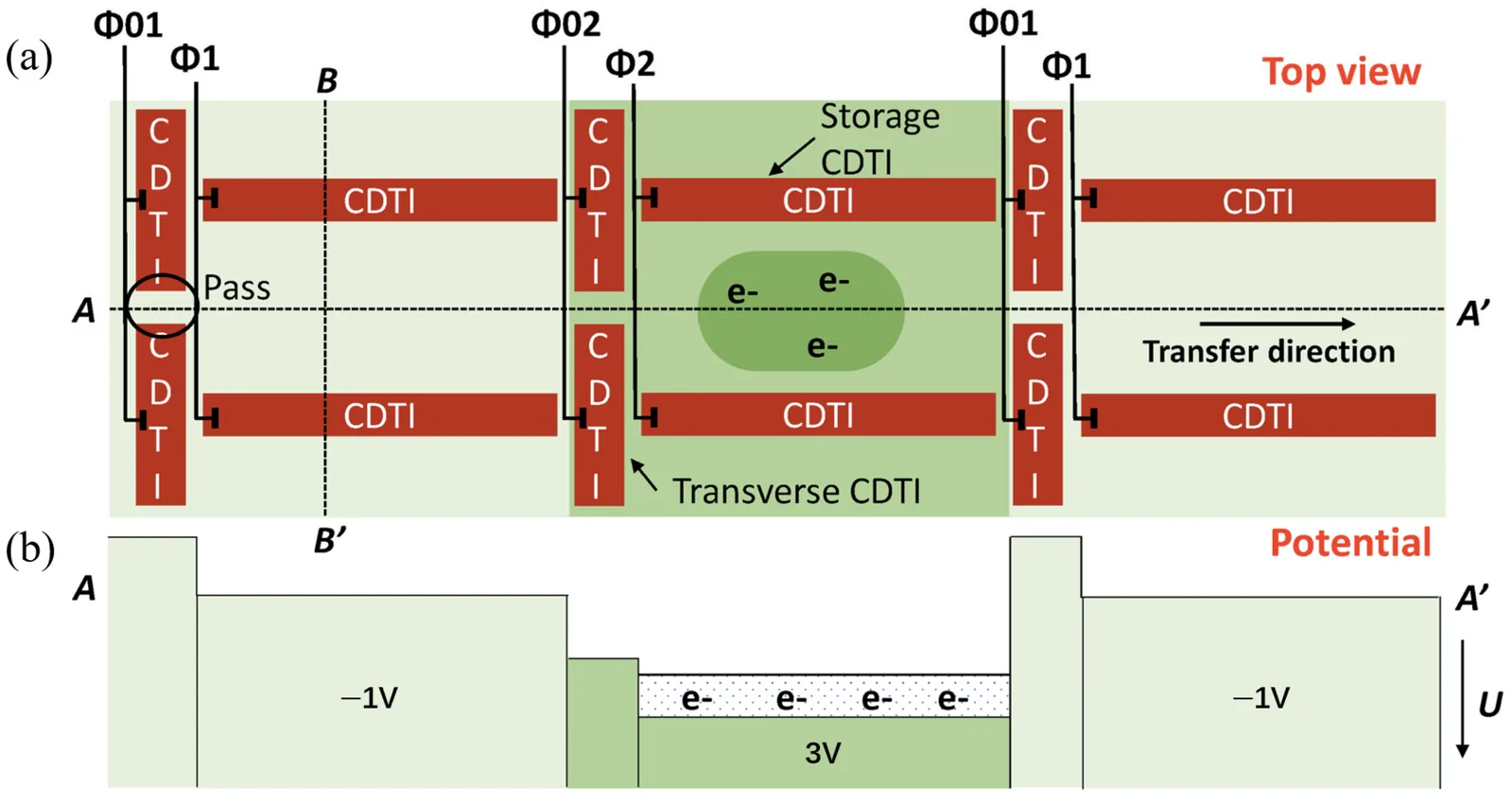
(**a**) Top schematic view of the CDTI phase register. (**b**) Schematic representation of the electrostatic potential profile along the transfer path. (Reproduced from Reference [61] with permission from O. Marcelot, et al., Sensors; published by MDPI, 2023).

In 2023, the CDTI is used in an EMCCD, which shows a charge transfer inefficiency (CTI) lower than 10^−4^ and a dark current of 0.11 nA/cm^2^, proving its great potential in reducing dark current and noise. The CDTI structure adopts a two-phase configuration comprising two types of CDTIs (Figure 6a): storage CDTIs aligned along the charge transfer path, and transverse CDTIs separated by a pass and oriented perpendicularly to the storage one. The transverse CDTI is designed to create a lower potential barrier between phases, enabling charge storage in one phase even when neighboring phases are biased at the same voltage (Figure 6b) and also can reduce optical and electrical crosstalk. By biasing the CDTI in inversion, passivation of defects at the Si-SiO_2_ interfaces along the CDTI edges is achieved, resulting in very low dark current generation.

In 2026, a CCD based on CDTI with 3 × 12 μm pixels achieves great improvement in dark current and CTI. The device is operated at 100 kHz and pulsewidth of 100 ns; the minimum CTI is 10^−5^ and the dark current is down to 21 pA/cm^2^ with acceptable reduction in FWC.

### 3.5. The Developments of the Material Sector

For fields such as spectral detection and astronomical observation that require strong near-infrared response, CCDs must achieve high near-infrared QE. With the improvement of spatial and spectral resolution in spaceborne hyperspectral imaging systems, the demand for pixel count continues to increase. If pixels cannot be miniaturized, the scale of the optical system will increase, making the design and fabrication of optical systems more difficult, while also significantly increasing system volume and weight, thereby making fabrication and launch costs difficult to bear. Therefore, improving detector QE through material modifications is a feasible approach to increase the amount of information acquired by the detection system. Since the all-silicon structure of conventional CCDs limits the device spectral response to the visible band, improvements at the material level have become a promising research direction.

**Figure 7:**
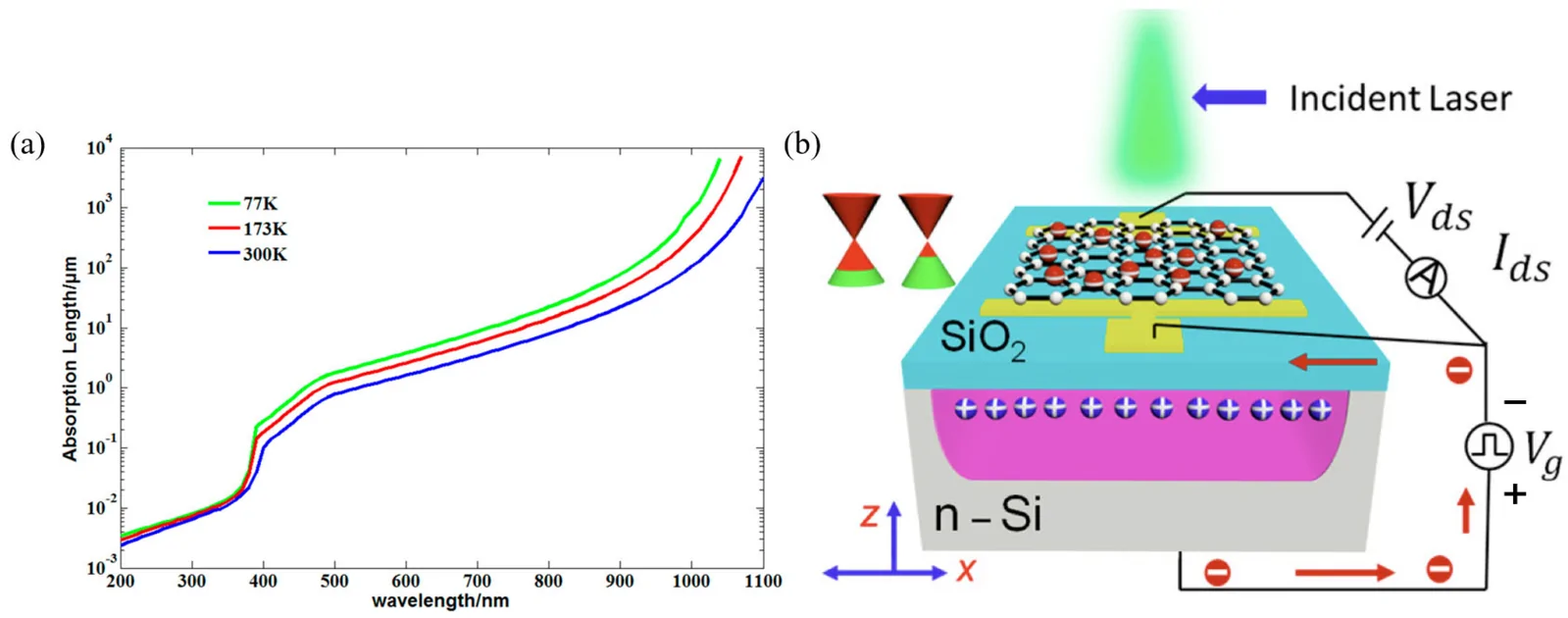
(**a**) Relationship between silicon absorption length and wavelength at temperatures of 77, 173, and 300 K (Reproduced from Reference [63] with permission from B. Hu, et al., Remote Sensing; published by MDPI, 2018). (**b**) 3D schematic of Graphene–Silicon CCD pixel (Reproduced from Reference [24] with permission from M. Ali, et al., Sensors; published by MDPI, 2022).

CCDs employing deep-depletion technology can achieve depletion depths of 200–300 μm or even higher, significantly improving near-infrared QE. Figure 7a shows the schematic diagram of light absorption at different wavelengths for substrates of different thicknesses. Therefore, for applications requiring both visible and near-infrared bands, deep-depletion silicon is an ideal choice [63,64,65,66]. Deep-depletion CCDs are fabricated with thicker silicon, enabling detection of deeper near-infrared wavelengths that can penetrate further into the silicon layer. Such deep-depletion silicon is typically also high-resistivity silicon, capable of operating at higher voltages and forming a thicker depletion region. In contrast, conventional depletion-mode CCDs, due to insufficient thickness, allow near-infrared wavelengths to pass through without being absorbed. A device known as the HiRho CCD [67] consists of an epitaxial layer grown on a high-resistivity silicon substrate via deposition processes. It can also achieve a relatively thick depletion region and exhibits high QE in the red spectral band.

Furthermore, due to the fact that the thickness of 2D materials is only a few atoms and they possess unique physical properties, they are expected to become an important material for driving the development of the semiconductor industry [68,69]. Some research groups have considered introducing new materials to broaden the applicability of CCDs. In 2017, MIT Lincoln Laboratory proposed a germanium-based semiconductor CCD detector for near-infrared and X-ray imaging [70]. In recent years, research teams at Zhejiang University have proposed using graphene and other 2D materials to achieve performance breakthroughs in CCDs, mainly focusing on improvements in charge readout and broadband spectral performance. These novel devices may not be limited by the scaling constraints encountered by traditional silicon-based devices during miniaturization. They proposed the Graphene–Silicon CCD (Figure 7b) and graphene charge-injection (GCI) photodetectors, which combine the advantages of CCD and CMOS technologies and enable detection and imaging to middle-infrared light regime [21,22,24,26]. The Graphene–Silicon CCD has a pixel size of 5 μm, and its readout mechanism differs from that of conventional CCDs. The field effect of graphene enables direct readout of charges in the potential well, avoiding charge crosstalk between adjacent wells in traditional CCDs. Moreover, charge readout and charge integration processes are relatively independent, meaning that readout operation does not affect charge accumulation, but graphene only serves the functions of charge readout and amplification in the Graphene–Silicon CCD, does not achieve significant beneficial effects in the near-infrared regime and infrared regime. A GCI photodetector consists of a silicon deep-depletion well for charge integration and incorporates a multilayer graphene layer to absorb infrared light, thereby extending its bandwidth into the mid-infrared regime. They also proposed a deep-depletion graphene field-effect coupled detector with excellent X-ray characterization performance. The graphene field-effect coupled device can maintain high responsivity and sensitivity over a broad spectral range including soft X-rays, ultraviolet, visible, and infrared bands, offering a viable strategy to monolithically integrate 2D materials into conventional solid-state imaging technology. The device, using a graphene channel, realizes carrier multiplication by exploiting strong silicon surface potential, instead of relying on traditional gate-to-gate coupling used in traditional EMCCDs. What’s more, a silicon-based molybdenum disulfide CCD structure is proposed in 2022 [23], which is divided into a photosensitive layer and a readout layer. The photosensitive layer is similar to that of conventional devices, consisting of silicon dioxide and a lightly doped silicon substrate; the readout layer consists of a graphene–MoS_2_–graphene heterojunction for real-time, non-destructive charge readout. This structure achieves both high responsivity and high readout speed with a relatively simple device architecture, and exhibits significantly lower static power consumption compared with graphene-channel devices, providing a new route for improving the performance of transition-metal-dichalcogenides-based photodetectors. At present, this technology is inferior to current commercial CCDs and it is still at the theoretical exploration stage, requiring further research and development. Table 3 compares the performance of several emerging sensors described above.

### 3.6. The Improvements in Manufacturing Techniques

As can be seen from Section 3.2, the backside-illuminated structure plays an important role in pixel miniaturization. To realize a backside-illuminated architecture, the silicon wafer used for growing the device is typically thinned from the backside, leaving only part of the epitaxial layer [71]. At this point, its mechanical strength is insufficient to maintain planarity, and, due to stress, the wafer may deform and warp. Therefore, before backside thinning, the front side of the CCD image sensor must first be bonded and fixed to a supporting substrate or glass substrate. Wafer bonding generally refers to directly connecting the regions of two wafers that need to be in contact using physical or chemical methods. The main wafer bonding processes currently include anodic bonding, direct bonding, and intermediate-layer bonding. Anodic bonding involves applying a certain pressure to bond the wafer and glass, then connecting a power supply and heating; under the action of an electric field and high temperature, dehydration occurs at the interface to form strong O-Si-O chemical bonds, completing the bonding. Direct bonding involves surface pretreatment of two silicon wafers followed by pre-bonding and thermal treatment, requiring extremely high surface quality of the silicon wafers. Intermediate-layer bonding uses a bonding interlayer to join the two wafers.

Backside thinning is commonly performed using mechanical thinning and wet etching [49]. Mechanical thinning first removes most of the substrate using a high-efficiency and uniform mechanical grinding process, followed by fine grinding once most of the substrate has been removed. The fine grinding wheel has finer abrasive grains and higher density; therefore, during mechanical grinding thinning, it introduces a shallower damage layer and lower surface roughness, although it still causes some damage to the silicon wafer. Wet etching processes introduce almost no defects, and by controlling the etching amount, defects generated during mechanical polishing can be effectively removed. HNA solution is a commonly used isotropic etchant in wet etching, and different mixing ratios lead to different etching rates for silicon with different doping concentrations. Therefore, an optimized etchant formulation can be designed so that the etching rates of the substrate and the epitaxial layer differ significantly, allowing the epitaxial interface to act as an etch-stop layer during the thinning process [72,73,74]. Due to surface non-uniformity after mechanical thinning and variations in wafer conditions during wet etching, inconsistent etching progress may occur among wafers, so equipment capable of controlling etching uniformity is also required.

**Figure 8:**
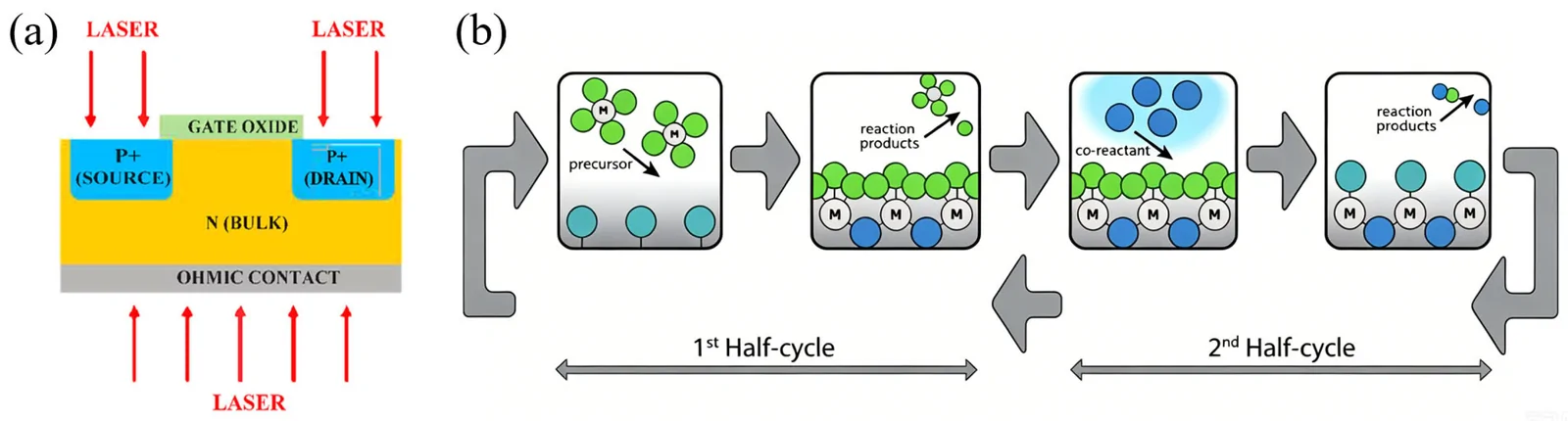
(**a**) Scheme of the laser-annealing process exploited to activate doping in the source and drain region and form ohmic contact (Reproduced from Reference [77] with permission from D., Arduino, et al., Materials; published by MDPI, 2023). (**b**) Atomic layer deposition process (Reproduced from Reference [82], © 2022, with permission from Elsevier).

After backside thinning, backside processing is still required due to the presence of a high density of interface states on the rear surface. Surface and boundary effects have a more pronounced impact on small pixels; if no proper passivation or anti-reflection treatment is applied to the backside, the photosensitive and charge collection regions are more susceptible to surface recombination losses [75]. Surface passivation methods include field-effect passivation and chemical passivation. Field-effect passivation is the establishment of an electrostatic field at the interface via charge accumulation, which reduces the surface minority carrier concentration [76], usually through a two-step process of ion implantation and annealing activation. To improve QE in the ultraviolet band, a very shallow junction depth is required, typically achieved using low-energy ion implantation. Laser annealing (Figure 8a) uses a flat-top beam to irradiate the device surface, generating a localized and instantaneous high temperature in the surface layer to activate dopant ions [77,78,79]. This approach offers significant advantages over traditional annealing methods by enabling precise control over the thermal energy distribution while minimizing unwanted thermal effects on adjacent materials.

Chemical passivation aims to suppress the density of surface states, which can be achieved through hydrogen annealing [80]. For example, using Al_2_O_3_ as a passivation material can prevent parasitic shunting. Atomic-layer-deposited Al_2_O_3_ provides excellent surface passivation for p-type, n-type, and highly doped p-type emitter surfaces, owing to its high negative fixed charge density, low interface state density, and low surface recombination velocity [49,81]. At the same time, as a dielectric material, Al_2_O_3_ carries negative fixed charges that repel negative charges on the silicon surface, inducing the formation of a hole-accumulation layer and thereby achieving field-effect passivation. Al_2_O_3_ thin films deposited by atomic layer deposition (ALD) are used, and Figure 8b shows a schematic diagram of the ALD process [82]. This passivation layer can be directly applied to the thinned substrate of backside-illuminated CCDs, thereby reducing dark current, improving response uniformity, and optimizing light collection. In addition, by further depositing a well-designed anti-reflection coating on top of the passivation layer, reflection losses can be further reduced and QE can be improved.

## 4. Summary and Outlook

Looking back at the development of CCDs over the past two decades, with advances in backside-illuminated structures, anti-blooming architectures, and microlens technologies, CCDs that meet the performance requirements of fields such as astronomy and scientific observation have now reached mainstream pixel size of around 10 μm, with some devices achieving 7 μm. The device with CCD-in-CMOS process has achieved a pixel size of 3.5 μm. However, CCD pixel miniaturization still faces numerous challenges. In current CCD fabrication processes, reducing pixel size leads to a decrease in potential well depth and charge capacity, resulting in reduced FWC and dynamic range. At the same time, because CCDs typically adopt highly doped multi-phase, multi-layer gate structures, the fabrication process is already complex, and further pixel scaling imposes even higher manufacturing requirements.

To address these challenges in pixel miniaturization, the third part of this review summarizes multiple approaches explored by researchers. These technological breakthroughs provide feasible pathways for continued scaling of image sensor pixels. The backside-illuminated structure is widely applied in the CCDs. For example, CCD47-10 and CCD47-20 both have backside-illuminated format. Microlenses and anti-blooming structures are widely used in CCD image sensors. But no solution has yet been achieved that simultaneously balances high QE, high CTE, low noise, and high yield. In terms of practical applications, particularly in aerospace, the Beijing Institute of Space Mechanics and Electricity has developed a spaceborne spectrometer based on an area CCD with a pixel size of 13 μm × 13 μm [83]. Furthermore, the Sentinel series satellites for Earth observation employ CCDs designed by Teledyne e2v. However, some performance-improving methods become significantly less effective once the pixel size is reduced to a certain extent [62] and new directions such as 2D material and CCD-in-CMOS process need further research. Considering practical requirements, small pixels may not be as practical as devices with larger pixels. Some methods improving the performance parameters of CCDs like maturing multi-CCD mosaic array technologies already exist, making the demand for pixel miniaturization relatively limited. In some applications requiring smaller image sensors, CMOS image sensors have already achieved pixel sizes down to 0.56 μm [84] or even smaller and the image quality of CMOS has been continuously improving and has now reached the level of CCDs in many application areas.

Although CCD pixel size is held back by fabrication constraints, physical limits, and practical requirements, CCDs still hold their ground in high-end scientific imaging, space exploration, and defense remote sensing, thanks to their strengths in high sensitivity, low noise, and high dynamic range. Moreover, the introduction of EMCCD technology and the combination of CMOS-compatible processes with 2D materials are creating fresh opportunities for CCD image sensors to advance across a wide spectral range. The future of CCD pixel miniaturization will continue to leverage its characteristics in current advantageous areas, pursuing research toward higher CTE, lower noise, faster readout speeds, and broader wavelength detection ranges, while maintaining existing image quality. The CCD-in-CMOS process represents a new development path that may serve as a significant driving force for advancing CCD miniaturization.

## Figures and Tables

**Table 1:** Performance comparison among different CCDs.

Parameters (Typical)	S12379	CCD290-99	CCD47-20	[16]	iXon Ultra 888	[11]
Type	Linear CCD	Full-frame CCD	Frame-transfer CCD	Interline-transfer CCD	EMCCD	CCD-in-CMOS
Pixel size	8 × 8 μm	10 × 10 μm	13 × 13 μm	7.4 × 7.4 μm	13 × 13 μm	3.5 × 3.5 μm
Active Pixels	2048	9216 × 9232	1024 × 1024	1920 × 1080	1024 × 1024	NR
FWC	NR	90 ke^−^	100 ke^−^	58 ke^−^	80 ke^−^	3 ke^−^
Conversion gain	21 μV/e^−^	7.5 μV/e^−^	4.5 μV/e^−^	22.4 μV/e^−^	NR	62.6 μV/e^−^
QE Max	~80% (25 °C)	>90% (−100 °C)	>75%	NR	>95%	>80%
Dark signal	30 e^−^/ms	4 e^−^/h (−100 °C)	60 e^−^/s	NR	2.5 × 10^−4^ e^−^/s (−85 °C)	NR
CTE	0.99999	0.999995	0.999999 (parallel) 0.999993 (serial)	NR	NR	>0.99998
Application scenarios	Machine vision; High-speed image readout	Astronomy	Spectroscopy; Scientific Imaging Medical Imaging	Complex Lighting Environment	Quantum imaging; Fast astronomy; Tomography; Single molecule detection	High-speed and large-FWC applications(in future)

**Table 2:** Technologies of pixel miniaturization and brief introduction to effects on parameters.

Technology	FWC	QE	Crosstalk	Noise	CTE
Backside-illuminated structure	N/A	Increase	N/A	Increase	N/A
Microlenses	N/A	Increase	Increase	N/A	N/A
Traditional anti-blooming structure	Decrease	N/A	Decrease	N/A	N/A
Capacitive deep trench isolation	Decrease	N/A	Decrease	Decrease	Increase (no clear improvements on smaller pixel)
Passivation	N/A	N/A	N/A	Decrease	N/A

**Table 3:** Performance comparison among different CCDs with new technologies.

Parameters	[11]	[62]	[24,26]	[23]
Technology	N/A	CDTI	Graphene–Silicon CCD	Silicon-based MoS_2_ CCD
Pixel size	3.5 × 3.5 μm	3 × 12 μm	5 × 5 μm	NR
Working frequency	>5 kHz	100 kHz	75 kHz	NR
Responsivity	NR	NR	6 × 10^4^ A/W	7.5 × 10^4^ A/W
Response time	NR	NR	~1 μs	70 ms
FWC	3 ke^−^	68 ke^−^	1.8Me^−^	NR
QE	>80%	NR	64% (532 nm)	NR
Dark current	13.7 nA/cm^2^	21 pA/cm^2^	NR	NR
CTE	>0.99998	>0.99999	0.924	NR

## Data Availability

No new data were created or analyzed in this study. Data sharing is not applicable to this article.
